# Supplementary material for: Vaccine-Induced Antibody Responses against SARS-CoV-2 Variants-Of-Concern Six Months after the BNT162b2 COVID-19 mRNA Vaccination
Source: Microbiol Spectr. 2022 Mar 9;10(2):e02252-21. doi: 10.1128/spectrum.02252-21 (PMC9045126; doi:10.1128/spectrum.02252-21)
Supplement: SUPPLEMENTAL FILE 1 — Supplemental material. Download SPECTRUM02252-21_Supp_1_seq4.pdf, PDF file, 0.2 MB [file spectrum02252-21_supp_1_seq4.pdf]

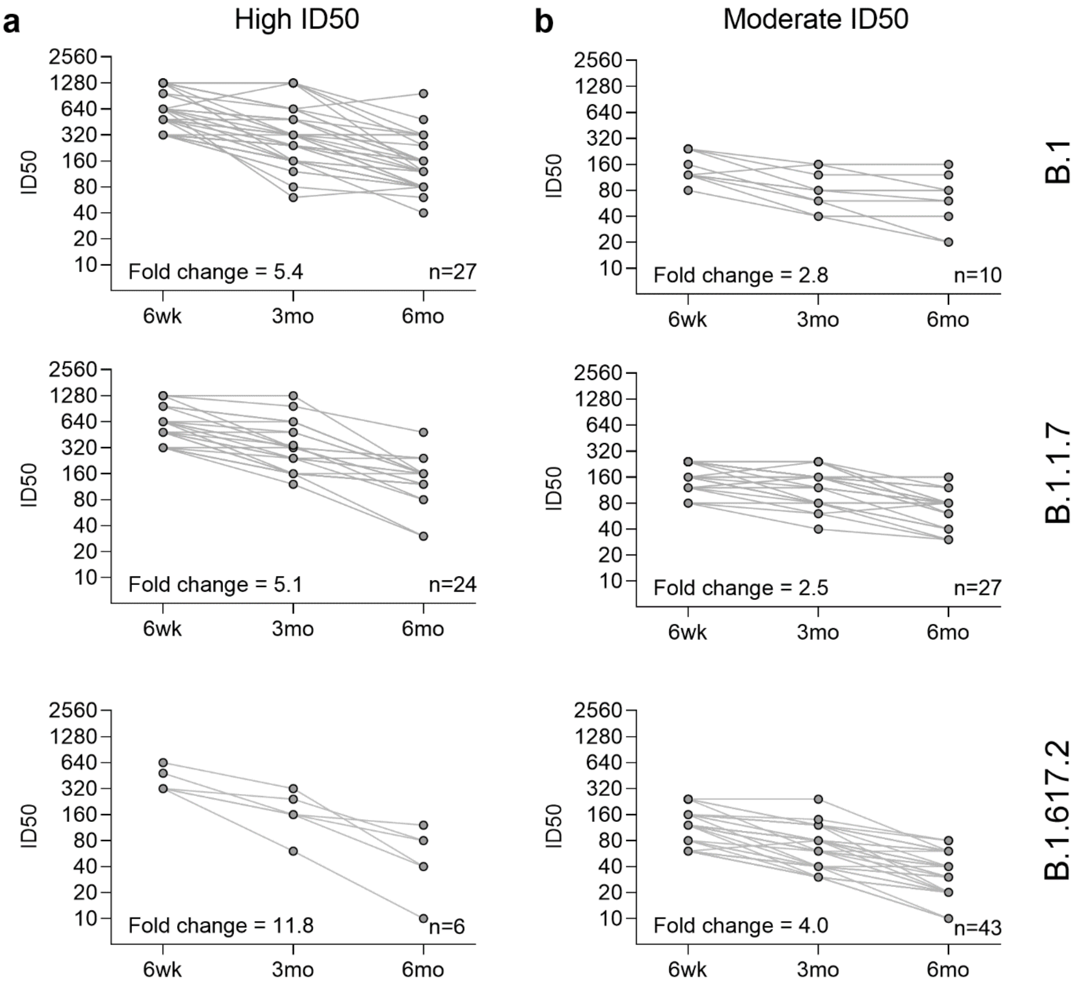

Supplementary Fig. 1 Vaccinees were grouped to low (ID50<60), moderate (ID50 60-320), and high (ID50>320) neutralization titer groups based on the neutralization titer at 6wk sample. Neutralization titers against D614G (B.1), Alpha (B.1.1.7), and Delta (B.1.617.2) were compared at 6 weeks, 3 months, and 6 months post vaccination. The fold changes between 6wk and 6mo samples were calculated. High neutralization titer group is shown on the left and moderate neutralization titer group on the right with individual trajectories for each sample. Low neutralization titer group is not shown due to no samples or low number of samples; B.1 n=0, B.1.1.7 n=1, and B.1.617.2 n=3.
